# Association between salivary pH and metabolic syndrome in women: a cross-sectional study

**DOI:** 10.1186/1472-6831-12-40

**Published:** 2012-09-08

**Authors:** Monique Tremblay, Diane Brisson, Daniel Gaudet

**Affiliations:** 1 Université de Montréal, Department of Medicine, ECOGENE-21 Clinical Research Center; Chicoutimi Hospital, 305 St-Vallier Street, Chicoutimi (Québec), Saguenay, G7H 5 H6, Canada

**Keywords:** Salivary pH, Cardiometabolic risk, Metabolic syndrome

## Abstract

**Background:**

The salivary flow rate is an important determinant of salivary pH. It is influenced by several metabolic syndrome (MetS) components as well as the menopausal status. The cluster of cardiometabolic risk factors that characterizes the MetS could be exacerbated following menopause. The objective of this study was therefore to document the association between salivary pH and MetS expression in women according to the menopausal status.

**Methods:**

In this cross-sectional study, unstimulated saliva collection was performed on 198 Caucasian women of French-Canadian origin of which 55 were premenopausal women (PMW) and 143 menopausal women (MW). Student’s t test, ANOVA and correlation analyses were used to assess the association between salivary pH and MetS components.

**Results:**

The salivary pH level was significantly correlated with several MetS covariates, namely triglycerides (TG), apolipoprotein B (apo B) and plasma glucose concentrations as well as waist circumference and the number of MetS components present in the whole sample and PMW only. Mean pH levels decreased as the number of MetS components increased (p = 0.004). The correlations between salivary pH and variables associated with MetS components tended to be stronger in PMW. The proportion of the variance (R^2^) of salivary pH explained by MetS-related variables in PMW, MW and the whole sample was 23.6% (p = 0.041), 18.1% and 17.0% (p < 0.001) respectively.

**Conclusions:**

The increasing prevalence of obesity calls for the development of new technologies to more easily monitor health status without increasing the burden of healthcare costs. As such, the salivary pH could be an inexpensive screening tool. These exploratory data suggest that salivary pH may be a significant correlate of the expression of MetS components. However, other studies with different populations are needed to confirm these findings before our observations lead to practical use in clinical settings.

## Background

The metabolic syndrome (MetS) is a cluster of interrelated common clinical disorders, including obesity, hyperglycemia, insulin resistance, dyslipidemia, increased apolipoprotein B (apo B) levels, inflammation and hypertension, associated with an increased risk of type 2 diabetes mellitus (T2DM) and cardiovascular disease (CVD) [1]. MetS has received worldwide attention in the past few years because of its prevalence, now ranging from 20% to 30% of the adult population of almost all western countries, rising in parallel with that of obesity [1]. It has also been shown that its prevalence increases significantly during the perimenopausal and early postmenopausal years, independently of known CVD risk factors [2]. This may be a direct result of ovarian failure or, alternatively, an indirect result of the metabolic consequences of central fat redistribution with estrogen deficiency [3]. In response to the increasing prevalence of obesity and associated disorders coupled with the aging of the western population, the MetS and the burden of its consequences should become even more frequent in the coming years. It thus becomes imperative to improve the development of preventive and therapeutic strategies to slow down MetS progression and reduce the risk of T2DM and CVD. However, because of the insidious development of the MetS, its early detection proves difficult. Indeed, most people affected by it ignore their condition [1]. Consequently, the MetS is often diagnosed at a late stage when signs and symptoms compel the affected person to seek medical care [4,5]. This situation limits preventive interventions and calls for new ways to carry out simple, early assessments of MetS expression without increasing the burden of healthcare costs. To meet this need, the measurement of salivary pH, which is readily accessible and inexpensive, could provide an interesting avenue.

The salivary flow rate is critical for the maintenance of whole body health. Saliva helps bolus formation by moistening food, protects the oral mucosa against mechanical damage, plays a role in preliminary digestion and has defense functions against pathogen microorganism [6]. The saliva flow rate is also a modulator of salivary pH. At low flow rate, less bicarbonate is released, and pH decreases [7]. The salivary flow rate varies widely between subjects [8,9]. Women have lower flow rates and seem to have more variation in salivary pH than men. Hormonal fluctuations during events like puberty, menstruation, pregnancy and menopause could explain those differences [10]. Salivary pH and flow rate are also affected by various MetS components, such as obesity, hypertriglyceridemia and hypertension [11-15]. Moreover, degenerative alterations in the acinar cells, which cause a decrease of the saliva flow rate and a diminution of salivary pH, are frequently observed among diabetic and dyslipidemic patients [11,12].

The aim of this study was to examine the association between salivary pH and MetS component expression in women, taking into account their menopausal status.

## Methods

### Subjects and clinical data

The sample used in this study was composed of 198 Caucasian women of French-Canadian origin followed at the Chicoutimi Hospital Lipid Clinic (Quebec, Canada). Two groups were formed according to the menopausal status: 55 premenopausal women (PMW) and 143 menopausal women (MW). Menopausal status was attributed to women who self-reported that their menses had stopped for at least 12 months without surgery or had occurred in the past 12 months but not in the last 3 months. The menopausal status was also attributed when the hormonal cycle arrest was diagnosed by the physician or was automatically attributed to all women of 50 years old or older who had not confirmed that they still had menses at the time of the interview. A dry mouth is more prevalent in climacteric women than in premenopausal ones [16]. Considering that the hypothalamic-pituitary-ovarian axis can affect saliva output and can be affected before a woman reaches menopausal status, the age limit of 50 was chosen in order to include perimenopausal women [17].

Anthropometric variables were measured according to validated procedures [18]. MetS components considered were: abdominal obesity (waist circumference >88 cm), high triglyceride (TG) level (> 1.7 mmol/L), low HDL-cholesterol level (<1.3 mmol/L), elevated blood pressure (≥130/85 mm Hg or diagnosed hypertension) and elevated fasting glucose level (>5.6 mmol/L or diagnosed T2DM) [1,19,20]. T2DM was defined according to the World Health Organization criteria as a 2-h glucose concentration ≥11.1 mmol/l following a 75 g oral glucose load, whereas an impaired glucose tolerance (IGT) state was characterized by a 2-h glucose concentration between 7.8 and 11.1 mmol/L [21]. All hormonal drugs were combined in a same unique covariate. The lipid-lowering drugs were also grouped and processed as user/non-user of medication. A written informed consent was obtained for all participants, and all clinical data were de-identified. The project was approved by the Chicoutimi Hospital Ethics Committee, in accordance with the Declaration of Helsinki.

### Biochemical analyses

Blood samples were obtained after a 12-h overnight fast into vacutainer tubes containing EDTA. The HDL subfraction was obtained after precipitation of LDL (d > 1.006 g/ml) in the infranatant with heparin and MnCl_2_[22]. Cholesterol, glucose and TG levels were measured by enzymatic essays on a Multiparity Analyser CX7 (Beckman, Fullerton, CA, USA) [23]. Plasma glycerol concentrations were measured with an Technicon RA-500 analyzer (Bayer Corporation), and enzymatic reagents were obtained from Randox (Randox Laboratories). Apo B levels were determined using nephelometry.

### Saliva collection and pH measurement

Although stimulated saliva is generally taken as the index of salivary function, whole unstimulated saliva collection was chosen for the purpose of this study because it is the greatest contributor to the total salivary output [9,16,24]. All saliva samples were collected at least 2 hours after any food intake or smoking, and the same day as blood samples. Unstimulated saliva was allowed to accumulate in the floor of the mouth, and the subject then spat it out into a test tube during 10 minutes. The pH of the saliva sample was measured with Accumet Basic AB 15 pH Meter (Ottawa, Canada), a 13-620-96 Micro pH electrode, 1.5” stem (127 mm) x 3 mm diameter with a pH range of 0 to 14 (Na + < 0.1 N) and a selectable resolution to 0.1, 0.01 or 0.001pH. The measurements were performed 3 times on each sample with a 0.01 resolution. The final result was the mean value of the measurements.

### Statistical analysis

Due to their skewed distribution, plasma TG, glycerol and apo B values were log_10_-transformed before analyses. Geometrical means are presented in Table 1. Differences in continuous variables were compared by either the Student’s t test or ANOVA. Categorical variables were compared using the Pearson χ^2^ statistic or Fisher’s exact test. Pearson’s correlations were performed to assess the relationship between MetS components and salivary pH. Fisher’s Z transformation was used to compare the correlation coefficients. All-in-one models multivariate regression analysis was constructed in order to investigate the relationship between salivary pH and covariates potentially affecting its concentrations, namely: age, smoking habits, waist girth, glycemia, TG and apo B levels. Use of medication as a covariate, including hormonal therapy and lipid-lowering drugs, did not change the results in the different models. All statistical analyses were performed with the SPSS package (release 11.0, SPSS, Chicago III).

**Table 1:** Subjects’ characteristics according to menopausal status

		**Pre-menopausal women**			**Menopausal women**		**p-value**
		***(n=55)***			***(n=143)***		
**Age (years)**	47.7	±	6.6	63.9	±	7.6	**<0.001**
*Range*	*24.0*	-	*52.0*	*39.0*	-	*82.0*	
**BMI (kg/m**^**2**^**)**	27.0	±	4.8	28.7	±	5.4	**0.040**
**Waist (cm)**	88.26	±	11.73	93.11	±	15.49	**0.037**
**Salivary pH**	6.64	±	0.34	6.70	±	0.30	NS
**Glycemia (mmol/L)**	5.68	±	2.08	5.76	±	1.62	NS
Glycerol (mmol/L) *	0.07	±	0.06	0.10	±	0.05	**<0.001**
**CT (mmol/L)**	5.79	±	1.36	6.05	±	1.05	NS
**HDL-C (mmol/L)**	1.35	±	0.48	1.41	±	0.33	NS
TG (mmol/L) *	1.36	±	3.86	1.68	±	1.65	**0.031**
Apo B (g/L) *^a^	1.01	±	0.28	1.08	±	0.21	NS
**Systolic blood pressure**	120.0	±	16.8	134.3	±	18.1	**<0.001**
**Diastolic blood pressure**	74.1	±	9.0	72.7	±	9. 2	NS
**T2DM (%)**		20.0			30.8		NS
**IGT or T2DM (%)**^**b**^		29.1			42.0		**<0.001**
**Exogenous hormone**							
**use (%)**		**0**			**54.5**		**<0.001**

Abbreviations **BMI**: body mass index; **CT**: cholesterol total; **HDL-C**: high-density lipoprotein cholesterol; **TG**: triglycerides; **Apo B**: apolipoprotein B; **T2DM**: type 2. Diabetes Mellitus; **IGT**: impaired glucose tolerance.

^a^ : n for PMW =54 and n for MW = 139.

^b^ : n for PMW =34 and n for MW = 74.

Mean ± SD. NS: p > 0.1.

*P-values were obtained after the log_10_-transpormation of the data, and geometric means are shown.

## Results

Subjects’ characteristics are shown in Table 1. The differences in mean values between PMW and MW reached the significance level (p < 0.05) for age, body mass index, waist circumference, plasma glycerol and TG levels, systolic blood pressure as well as the proportion of subjects under exogenous hormone therapy. In addition, the percentage of glucose intolerance in MW was significantly increased as compared to PMW (p <0.001): the percentage of T2DM in MW showed a 50% non-significant increase when compared to PMW.

**Figure 1:**
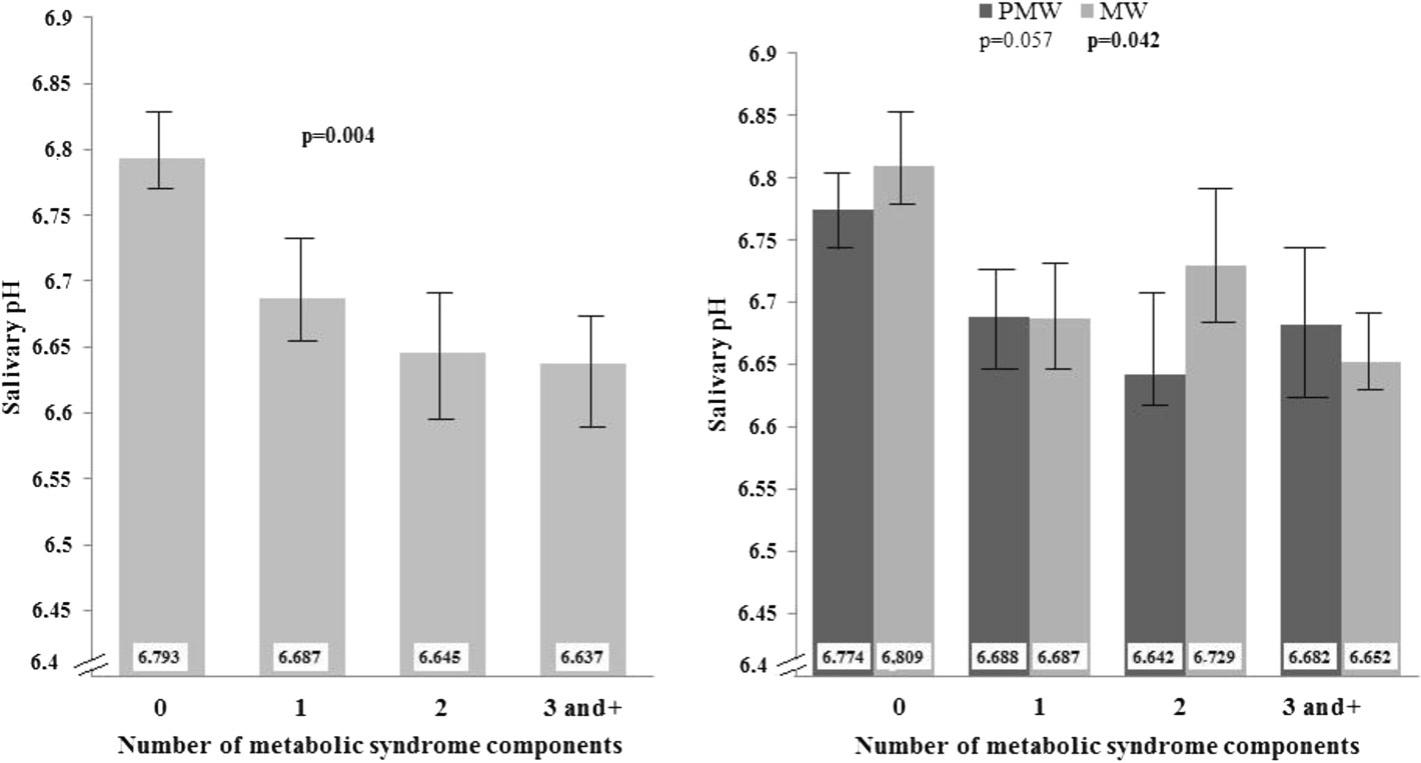
**Mean salivary pH according to the number of metabolic components taking into account the effect of age and smoking habits in the whole sample as well as among groups of PMW and MW.** The MetS elements considered are: abdominal obesity, dyslipidemia (high TG and low HDL-C levels), hyperglycemia and hypertension.

As shown in Figure 1, mean pH values decreased as the number of MetS components increased from 0 to ≥3 in the whole sample (p = 0.004), after adjusting for age and smoking habits. The results remained significant even when hormonal therapy was included as a covariable. The same trend was observed in both PMW and MW (respectively p = 0.057 and 0.042). There was a significant interaction effect between the number of MetS components and the menopausal status on the salivary pH (p = 0.001).

Significant correlations (Table 2) were noted between salivary pH and various MetS components. Plasma glucose, TG and apo B levels as well as the number of MetS components were significantly correlated to salivary pH in the whole sample and PMW, even after adjustment for age. The correlation coefficient between apo B and salivary pH was significantly higher among PMW as compared to MW (p = 0.04). The correlation between plasma glucose and salivary pH also tended to be higher among PMW (p = 0.06). The correlation between salivary pH and waist circumference and the number of MetS elements reached the significance level in the whole group and MW when corrected for age. Finally, plasma TG levels were significantly correlated to the salivary pH among all groups of women, even when the results were corrected for age.

**Table 2:** Correlation between salivary pH and variables associated with the metabolic syndrome

				**Age-adjusted**		
	**All women**	**PMW**	**MW**	**All women**	**PMW**	**MW**
n =	198	55	143	198	55	143
TG*						
r	-0.224	-0.300	-0.211	-0.294	-0.292	-0.289
*p*-value	**0.001**	**0.026**	**0.011**	<**0.001**	**0.034**	**0.001**
Apo B*						
r	-0.251	-0.458	-0.159	-0.293	-0.448	-0.149
*p*-value	<**0.001**	<**0.001**	0.062	<**0.001**	**0.001**	0.082
**Glycemia**						
**r**	**-0.163**	**-0.320**	**-0.081**	**-0.198**	**-0.304**	**-0.137**
*p-value*	**0.022**	**0.017**	0.336	**0. 006**	**0.027**	0.112
**Waist**						
r	-0.142	-0.167	-0.159	-0.168	-0.166	-0.184
*p*-value	**0.046**	0.224	0.060	**0.020**	0.234	**0.031**
**Systolic BP**						
r	0.047	-0.057	0.046	-0.052	0.023	-0.073
*p*-value	0.515	0.682	0.586	0.473	0.870	0.398
**Diastolic BP**						
r	-0.098	0.021	-0.140	-0.093	0.034	-0.122
*p*-value	0.173	0.880	0.096	0.201	0.809	0.156
**HDL-C**						
r	0.091	0.048	0.110	0.089	0.075	0.149
*p*-value	0.201	0.728	0.191	0. 222	0.596	0. 083
**MetS**						
r	-0.171	-0.283	-0.144	-0.238	-0.261	-0.214
*p-*value	**0.016**	**0.037**	0.086	**0.001**	0.059	**0.012**

**All correlation analyse are bivariate. TG**: triglycerides; **Apo B**: apolipoprotein B; **PMW**: premenopausal women; **MW**: menopausal women; **HDL-C**: high density lipoprotein-cholesterol; **MetS**: number of metabolic syndrome components. The MetS elements considered are: abdominal obesity, dyslipidemia (high TG and low HDL-C levels), hyperglycemia and hypertension.

*Log_10_-transformed.

As shown in Table 3, the proportions of variance of salivary pH explained by age and MetS-related variables were 23.6 (p = 0.041), 18.1 and 17.0 (p < 0.001) for the PMW, MW and the whole sample, respectively. Although R^2^ tended to be higher in the PMW group, the difference was not statistically significant. With the exception of age, the only variables to have a significant effect in the multivariate model were TG in MW (p = 0.032), and apo B in PMW (p = 0.021) and the whole sample (p = 0.007). In the multivariate analysis, age reached the significance level in MW and the whole group, but not in PMW.

**Table 3:** All–in-one models multivariate regression analyses of the relation between salivary pH and metabolic syndrome elements

	**Salivary pH**		
	**PMW**	**MW**	**Whole Sample**
*R*^*2 (%)*^	***23.6***	***18.1***	***17.0***
*p-value*	***0.041***	***<0.001***	***<0.001***
**TG**			
*B*	***-0.117***	***-0.262***	***-0.167***
*Standard error*	*0.176*	*0.121*	*0.097*
*t*	*-0.663*	*-2.164*	*-1.725*
*p*-value	*0.511*	***0.032***	*0.086*
**Apo B**			
*B*	***-1.207***	***-0.173***	***-0.658***
*Standard error*	*0.504*	*0.309*	*0.243*
*t*	*-2.397*	*-0.559*	*-2.704*
*p*-value	***0.021***	*0.577*	***0.007***
**Glycemia**			
*B*	***-0.022***	***0.002***	***-0.013***
*Standard error*	*0.28*	*0.017*	*0.014*
*t*	*-0.764*	*-0.104*	*-0.958*
*p*-value	*0.449*	*0.917*	*0.339*
**Waist**			
*B*	***0.004***	***-0.002***	***-0.001***
*Standard error*	*0.005*	*0.002*	*0.002*
*t*	*0.730*	*-1.197*	*-0.896*
*p*-value	*0.469*	*0.234*	*0.371*
**Age**			
*B*	***0.003***	***0.013***	***0.008***
*Standard error*	*0.007*	*0.003*	*0.002*
*t*	*0.455*	*4.059*	*3.839*
*p*-value	*0.651*	***<0.001***	***<0.001***
**Smoking habits**			
**B**	***-0.017***	***0.004***	***-0.014***
**Standard error**	*0.057*	*0.027*	*0.024*
**t**	*-0.289*	*0.146*	*-0.594*
**p-value**	*0.774*	*0.884*	*0.553*

**TG**: triglycerides; **Apo B**: apolipoprotein B; **PMW**: premenopausal women; **MW**: menopausal women.

*Log10-transformed.

## Discussion

In our study, we found a significant correlation between salivary pH and the expression of MetS components expression among women. To the best of our knowledge, this is the first study investigating the association between the MetS and salivary pH. Although numerous studies have shown correlations between serum and saliva levels for a wide range of components, none has studied saliva from this simple perspective [9,11,12,25-28].

The correlation between salivary pH and MetS component expression remained significant even after the inclusion of age as a covariate but tended to be stronger among PMW. This could be explained by the facts that aging is an important risk factor for the MetS and menopause is often associated with additional metabolic alterations [2,29,30]. Moreover, the role of hormones in several metabolic processes suggests that the onset of menopause may influence the relationship between salivary pH and MetS component expression. Estrogen deficiency may notably influence salivary flow rates by indirect pathways. Dyslipidemia, diabetes and hypertension have been related to a decrease of the salivary flow, and their simultaneous presence could act as a confounding factor [9,16,24].

Menopause, as a risk factor for almost all components of the MetS [31], may put a woman at risk of developing salivary dysfunction. In addition, other disorders associated with aging, including but not limited to obesity [31], rheumatoid arthritis and fibromyalgia [32], depression [33] and nutritional deficiencies [34], could also reduce the salivary function. The associated permanent histological changes in the salivary glands they can trigger may also explain the differences we tended to observe between PMW and MW in the present study [35].

Among the different variables associated with the MetS, TG is the most significantly related to salivary pH, followed by apo B and glycemia. Our results are therefore consistent with those of previous studies that have shown associations between salivary dysfunction and high plasma lipid levels, particularly hypertriglyceridemia and increased apo B concentrations [36-38]. However, plasma levels of apo B and TG are not independently related to salivary pH. Their relations with salivary pH vary when other covariates are added to the models (Table 3). Hypertriglyceridemia is part of a complex network of interrelated metabolic abnormalities that act as confounding factors when multivariate analyses are used [39]. This could contribute to explain the decrease of the effect of hypertriglyceridemia in the multivariate analysis. The significant result of apo B observed in MW may be explained by a redistribution of body fat to the abdominal region. Such a distribution is often associated with both menopause and increased TG levels [40].

Many studies have found that the saliva flow rate and pH are related to the level of glycemic control, particularly in the presence of a severely impaired control of blood glucose [41]. There is evidence that adverse hormonal, microvascular and neuronal changes in poorly controlled diabetes could contribute to salivary gland hypofunction [11,24,42-46]. The present study indicates that differences in salivary pH could be linked with glucose control, particularly in PMW. In addition, studies have reported conflicting results on the relationship between salivary pH and hypertension or hypertensive therapy [24,47-49]. Our observations are therefore consistent with those of studies that have found no difference between normal and hypertensive subjects. Finally, considering that a BMI over 25 kg/m^2^ in adults and obesity in childhood have been linked to hyposalivation [31,50], pH changes could also be driven by the increase in waist circumference frequently associated with a rise in plasma TG and apo B concentrations. This is consistent with the results of the univariate analysis, while no significant effect of the waist circumference on the salivary pH was observed in the multivariate models. The lack of significant association between age and salivary pH among PMW could be due to the combination of various factors including the small size of the group, the smaller age-range and the lower variability of the sample dispersion.

Our study has some limitations. Because of the cross-sectional nature of the study, confounding factors such as medication or permanent alterations in salivary glands were not taken into consideration. Also, we didn’t have information about the percentage on which subjects had undergone a hysterectomy/bilateral oophorectomy or where late perimenopausal. Moreover, this study does not give any information about the potential causal pathway affecting the salivary function. As an individual’s flow rate and salivary pH remain relatively constant during the different life stages, salivary gland hypofunction is commonly associated with concomitant diseases and daily use of drugs. Thus, a longitudinal study design with disease or medication pre-test and post-test should be more conclusive. Finally, our study should be replicated in larger samples of women with various risk levels of MetS components to obtain the real positive (PPV) and negative predictive values (NPV) as well as specificity and sensitivity values. However, despite its limitations, our study emphasized the important association between systemic and oral health. In the near future, further scientific advances in salivary diagnosis could lead dentists to be more involved in diagnosis and monitoring of MetS components.

## Conclusion

In the context of the increasing prevalence of obesity and the MetS, efforts may need to be directed at the identification of new, simple, non-invasive and inexpensive screening tools to improve preventive strategies without increasing the burden of healthcare costs. In this pilot study, the salivary pH appeared as a possible correlate of MetS component expression. However, further studies are needed to confirm our findings. They should include more subjects from various populations in order to develop our observations into a definitive methodology to monitor MetS components onset and progression utilizing salivary pH.

## Competing interests

The authors declare that they have no competing interests.

## Authors’ contribution

MT conceived the study design, performed the data analysis/interpretation and wrote the manuscript. DB and DG conceived the study design and revised the manuscript. All authors have read and approved the final manuscript.

## Pre-publication history

The pre-publication history for this paper can be accessed here:

http://www.biomedcentral.com/1472-6831/12/40/prepub
